# Cost-effectiveness analysis of pembrolizumab plus chemotherapy as first-line therapy for extensive-stage small-cell lung cancer

**DOI:** 10.1371/journal.pone.0258605

**Published:** 2021-11-15

**Authors:** Qiao Liu, Chongqing Tan, Lidan Yi, Xiaomin Wan, Liubao Peng, Jianhe Li, Xia Luo, Xiaohui Zeng

**Affiliations:** 1 Department of Pharmacy, The Second Xiangya Hospital of Central South University, Changsha, Hunan, People’s Republic of China; 2 Department of Nuclear Medicine/PET Image Center, The Second Xiangya Hospital of Central South University, Changsha, Hunan, People’s Republic of China; West China hospital, Sichuan University, CHINA

## Abstract

**Background:**

The phase III KEYNOTE-604 study confirmed the benefit of pembrolizumab combined with chemotherapy in the first-line treatment of extensive-stage small-cell lung cancer (ES-SCLC). Taken into account the clinical benefits of pembrolizumab and its high cost, this study aimed to assess the cost-effectiveness of adding pembrolizumab to standard first-line etoposide-platinum (EP) for patients with ES-SCLC from the US payer perspective.

**Methods:**

A Markov model was developed to compare the cost and quality-adjusted life-year (QALY) of pembrolizumab plus EP and placebo plus EP over a 10-year time horizon. Clinical efficacy and safety data were pooled from the KEYNOTE-604 trial. Utilities were obtained from published resources. Costs were mainly collected from Medicare in 2020. Sensitivity analyses were performed to examine the robustness of our model.

**Results:**

Adding pembrolizumab to standard first-line EP resulted in the better effectiveness than EP chemotherapy alone for ES-SCLC by 0.22 QALYs. Pembrolizumab plus EP was dominated economically by placebo plus EP, leading to an incremental cost-effectiveness ratio (ICER) of $334,373/ QALY. Deterministic sensitivity analyses indicated that the uncertainty in model parameters exerted no substantial effect on our results. Probability sensitivity analysis indicated that probabilities for pembrolizumab plus EP being cost-effective within a wide range of willingness to pay were modest.

**Conclusion:**

From the US payer perspective, the first-line treatment for ES-SCLC with pembrolizumab plus EP was not cost-effective compared with placebo plus EP. Although pembrolizumab combination chemotherapy was beneficial to the survival of ES-SCLC, price reduction may be the necessary to improve its cost-effectiveness.

## Introduction

Small-cell lung cancer (SCLC) is a highly malignant pulmonary tumor and characterized by high proliferation, great invasion, and priority of early distant metastases [1, 2]. Extensive-stage SCLC (ES-SCLC) approximately composes 2/3 of all diagnosed cases of SCLC [3], Although the majority of ES-SCLC occurs mutation of tumor protein p53 (TP53) (90%) and retinoblastoma1(RB1) (65%), the target driven gene is not clear, which leads to limited progression in the treatment of ES-SCLC [4]. In the pre-immunotherapy era, platinum based (carboplatin or cisplatin) etoposide chemotherapy has always been the standard-of-care first-line treatment for ES-SCLC [5–7], which is associated with poor outcomes [a 5-year survival rate of 6%-7% and median overall survival (OS) of approximately10 months] [8, 9]. More effective first-line therapy is urgently needed to improve patients’ clinical prognosis.

In recently years, immune checkpoint inhibitors (ICIs) have demonstrated promising antitumor activity in patients with ES-SCLC, including as first-line treatments [10–12]. Pembrolizumab is a humanized IgG4 monoclonal antibody that targets PD-1 pathway and restores T-cell immune activity [13]. In October 2016, pembrolizumab becomes the first PD-1 ICI approved for first-line use for patients with non-small cell lung cancer (NSCLC) [14]. Shortly afterward, the US Food and Drug Administration (FDA) has successively approved new indications for pembrolizumab, either as monotherapy or in combination with platinum and pemetrexed, but still confined to patients with advanced or metastatic NSCLC [14, 15]. Shortly thereafter in June 2019, the FDA granted accelerated approval for pembrolizumab as third-line or later therapy for patients with metastatic SCLC due to its durable response rate and a manageable safety profile [16], which marks its position in the field of SCLC. Recently, a phase III KEYNOTE-604 clinical trial assessed the efficacy and safety of adding pembrolizumab to standard first-line etoposide-platinum (EP) for ES-SCLC [12]. The results demonstrated that pembrolizumab plus EP significantly extended progression-free survival (PFS) (hazard ratio [HR], 0.75; 95% CI, 0.61 to 0.91; P = 0.0023), while also prolonged OS (HR, 0.80; 95% CI, 0.64 to 0.98; P = 0.0164) than EP chemotherapy alone [12]. Besides, safety profiles of pembrolizumab plus EP were as expected, with no new or unexpected toxicities observed [12]. These data support the value of pembrolizumab plus EP as first-line treatment in this historically difficult-to-treat cancer.

Give that the new efficacy evidence for pembrolizumab and its high cost, cost-effectiveness analysis comparing pembrolizumab combined standard first-line chemotherapy EP and EP alone was worth discussing. Previous study suggested that adding ICIs to standard first-line chemotherapy may not be considered as cost-effective choices for ES-SCLC [17, 18]. However, the cost-effectiveness of the most recently reported first-line ICI option-pembrolizumab, has yet to be investigated. Hence, we are interested in exploring whether this new combination therapy provide clinical benefit at an acceptable cost for patients with ES-SCLC from the US payer perspective.

## Materials and methods

### Simulation model

Clinical efficacy and safety data were collected from the results of the randomized, double-blind, phase III KEYNOTE-604 study (ClinicalTrials.gov Identifier: NCT03066778), this economic evaluation used no individual patient-level data to inform the model. As a result, this study was exempted from the approval of the institutional research ethics board.

Using TreeAge Pro 2018 software (TreeAge, Williamstown, Massachusetts), we established a Markov model to evaluate the long-term cost and effectiveness for patients with previously untreated ES-SCLC from the US payer perspective. Based entirely on the KEYNOTE-604 clinical trial [12], two first-line strategies were compared in our model, referred to as (1) pembrolizumab plus EP and (2) placebo plus EP. The pembrolizumab plus EP group received four doses of pembrolizumab and EP, followed by maintenance of pembrolizumab up to 35 doses. The placebo plus EP group only received up to four doses of EP. With regard to the full trial population receiving treatment, 71.1% (317 of 446) were treated with carboplatin, and 28.9% (129 of 446) were treated with cisplatin.

After the failure of first-line treatments, as reported in the KEYNOTE-604 trial, over half of the patients were treated with subsequent therapies (52.9% in the pembrolizumab plus EP group; 65.5% in the placebo plus EP group). Nivolumab plus ipilimumab, etoposide, topotecan and irinotecan were the common subsequent therapies. Following the recommendations of the National Comprehensive Cancer Network (NCCN) Guidelines, supportive care was provided to patients who did not receive subsequent therapy [19]. **S1 Table** provides the detailed information on first and second-line treatment regimens.

**Fig 1:**
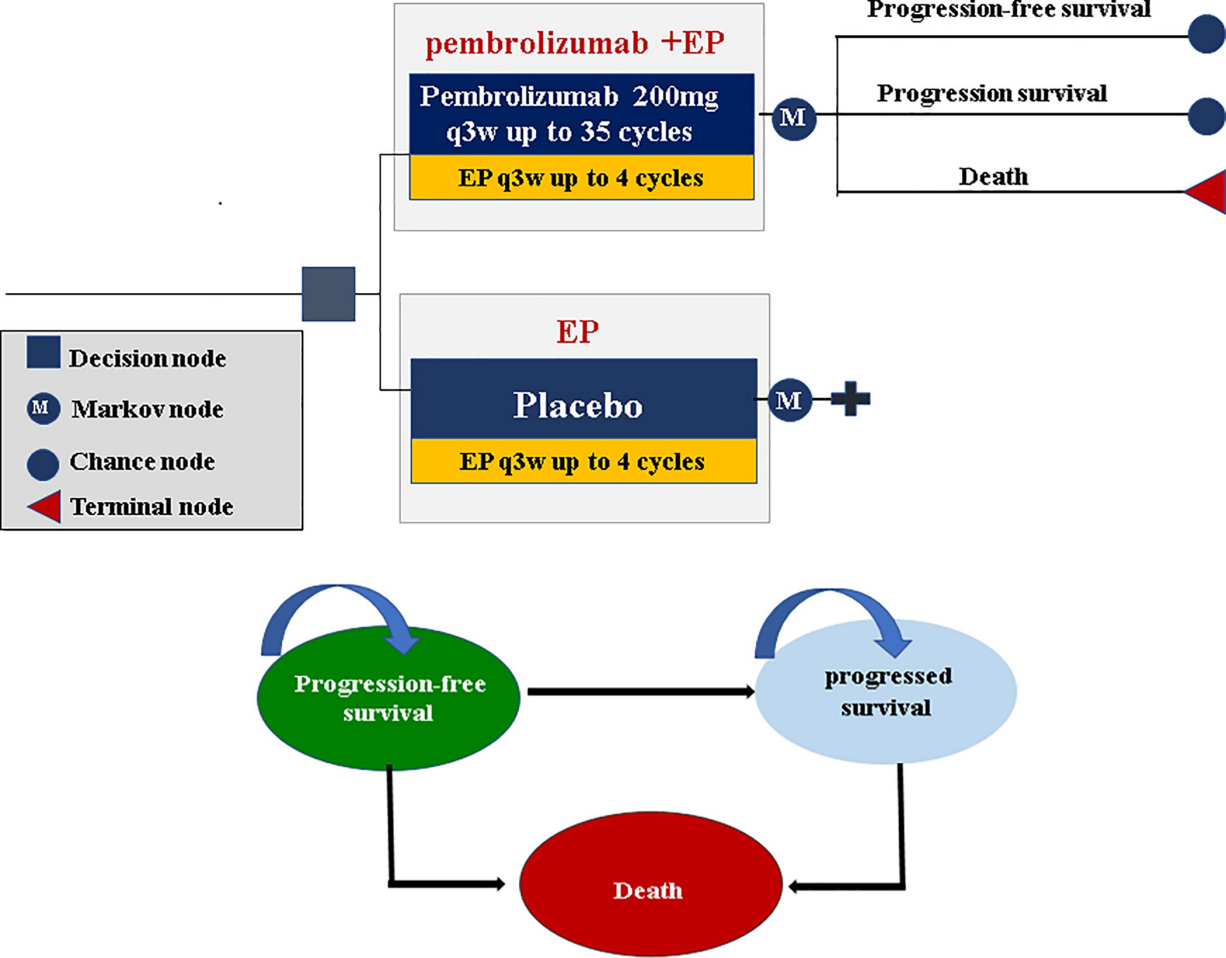
Markov model simulating outcomes for the patients with ES-SCLC in KEYNOTE-604 trial. The model consists of three health states: progression-free survival, progressed disease and death. All patients entered the model in the progression-free survival state and were randomly assigned to receive the two first-line treatment. During each Markov cycle, patients could move to another health state according to transition probabilities. ES-SCLC: extensive-stage small-cell lung cancer; EP: etoposide-platinum.

Our model consisted of three mutually exclusive health states including progression-free survival (PFS), progressed survival (PS), and death (Fig 1). All patients entered the model in the PFS state and could move to another health state according to transition probabilities. Patients in each heath state were assigned a certain health utility and corresponding treatment, incurred a certain medical cost and health effect [quantified in the form of quality-adjusted life years (QALYs)]. In this analysis, both cost and quality-adjusted life year (QALY) were discounted at an annual rate of 3%. Based on a consideration of therapeutic schedules and expected overall survival of ES-SCLC, a Markov cycle length of 3 weeks and a 10-year time horizon were set in our model. The summary outcome of our model was the incremental cost-effectiveness ratio (ICER), calculated as the incremental per additional QALY gained and compared with a willingness-to-pay (WTP) threshold of $100,000 per QALY [20].

### Survival and health state utilities

The Kaplan–Meier (KM) curves of OS and PFS reported in the KEYNOTE-604 trial were used for estimating the transition probability of death and disease progression from ES-SCLC treated with first-line pembrolizumab plus EP and placebo plus EP [12]. Firstly, OS data points for the first 33 months and PFS data points for the first 21 months were extracted from the OS and PFS KM curves using GetData Graph Digitizer software package (version 2.25; **http://www.getdata-graphdigitizer.com/index.php**), and then these data points were fitted and extrapolated by survival functions using R software (version 3.3.1, **http://www.r-project.org**). Secondly, using Akaike information criterion (AIC) and Bayesian information criterion (BIC), the goodness-of-fit of four commonly used parametric survival distributions were tested, including exponential, Weibull, log-normal and log-logistic. Thirdly, in view of lower AIC and BIC values indicating better fit, log-logistic distribution was chosen for this analysis (**S2 Table** detailed the AIC and BIC values). Finally, the log-logistic distribution parameters, theta (θ) and kappa (γ), were computed by R language (Table 1). To validate our model, the modeled survival curves were compared with the investigated KM curves (S**1**–S**4** Figs).

**Table 1:** Clinical inputs to the models.

Parameter	Input values	Ref
Log-logistic OS survival model		
Pembrolizumab plus EP	θ = 0.008564, γ = 1.719092	[12]
Placebo plus EP	θ = 0.002564, γ = 2.260147	[12]
Log-logistic PFS survival model		
Pembrolizumab plus EP	θ = 0.007161, γ = 2.510618	[12]
Placebo plus EP	θ = 0.000417, γ = 4.150059	[12]
Proportion of carboplatin treatment	0.711	[12]
Proportion of cisplatin treatment	0.289	[12]
Proportion of subsequent therapy		[12]
Pembrolizumab plus EP	0.529	[12]
Placebo plus EP	0.655	[12]
AE incidence of Pembrolizumab plus EP		
Neutropenia	0.435	[12]
Anemia	0.157	[12]
Thrombocytopenia	0.139	[12]
Pneumonia	0.067	[12]
AE incidence of placebo plus EP		
Neutropenia	0.408	[12]
Anemia	0.152	[12]
Thrombocytopenia	0.112	[12]
Pneumonia	0.045	[12]

OS: overall survival; PFS: progression-free survival; EP: etoposide-platinum; AE: adverse event.

The time-dependency transition probabilities of death from ES-SCLC were calculated based on the survival function of the log-logistic distribution:

$$tp_{die}(t_{u})=1-\frac{1+\exp(\theta ){\left(t-u\right)}^{k}}{1+\exp(\theta )t^{k}}$$

Accordingly, the time-dependency transition probabilities of PS from ES-SCLC were calculated as follow:

$$tp_{pfs}(t_{u})=\frac{1+\exp(\theta ){\left(t-u\right)}^{k}}{1+\exp(\theta )t^{k}}/(1-tp_{die})$$

Where the *u* was the Markov cycle, and *t* was calculated as integer multiples of the Markov cycle.

As information on quality-of-life data was not published as a part of the KEYNOTE-604 trial results, model inputs for health utility values were therefore sourced from EuroQOL-5D (EQ-5D) 3-level utility data of patients enrolled in the KEYNOTE-189 trial [21], because this trial focused on pembrolizumab combined platinum-based chemotherapy exactly as the KEYNOTE-604 trial [12, 22]. All information regarding heath utility values used in the model was provided in **S3 Table**.

### Costs

The collection of direct medical costs in our analysis were carried out from the three aspects: regimen related costs, adverse event (AE) management costs and other disease management costs.

Regimen related costs involved drug acquisition and administration costs. The cost of each drug was calculated on the basis of October 2020 Average Sales Price Drug Pricing Files (version updated October 3, 2020) form the U.S. Centers for Medicare & Medicaid Services (CMS) [23]. In calculating the dosage amounts, a base case patient with a body weight of 70.32kg, a body surface area of 1.79m^2^ and creatinine clearance rate of 70ml/min was assumed in our model [18, 24]. Drug administration costs were obtained though Physician Fee Schedule Look-Up Tool form CMS [25]. Statistics of adverse event management costs were based on the Healthcare Cost and Utilization Project using diagnosis Code selection for ICD-10 [26]. Grade 3 or higher serious AEs with an incidence of more than 3% were considered in the model. Other disease management costs, including outpatient follow-up visit costs, supportive care costs and death-associated costs, were derived from previously published articles [18, 24]. All information regarding costs used in the model was listed in **S3 Table**.

### Sensitivity analysis

To test the robustness of our model with respect to uncertainty in model parameters, one-way deterministic sensitivity analyses (DSA) and probabilistic sensitivity analysis (PSA) were performed. In DSA, the influence of model parameters varying individually on the cost-effectiveness results were assessed. The plausible ranges of model parameters were derived from publish studies whenever available or ±20% of base-case values. In PSA, the influence of model parameters varying simultaneously on the cost-effectiveness results were assessed. Model parameters were randomly sampled from the prespecified distributions, which followed the recommendations of the ISPOR-SMDM Modeling Good Research Practice Working Group [27]. We performed PSA using a Monte Carlo simulation with 1,000 iterations to generate 1,000 ICER estimates for the two competing first-line treatments. Parameter ranges and distributions use in the DSA and PSA were detailed in Table 2.

**Table 2:** Model parameters: Baseline values, ranges, and distributions for sensitivity analysis.

parameters	value	Ranges	Distribution	Ref
**Costs**				
**Regimen related costs**				
Pembrolizumab price/mg	49.39	39.51–59.27	Gamma (100,0.2.025)	[23]
Etoposide price/mg	1.51	1.21–1.81	Gamma (100,66.353)	[23]
Carboplatin price/mg	0.06	0.05–0.07	Gamma (100,1732.502)	[23]
Cisplatin price/mg	0.19	0.15–0.23	Gamma (100,533.049)	[23]
Nivolumab price/mg	27.81	22.25–33.37	Gamma (100,3.596)	[23]
Ipilimumab price/mg	153.13	122.50–183.75	Gamma (100,0.653)	[23]
Topotecan price/mg	414.63	331.71–497.56	Gamma (100,0.241)	[23]
Irinotecan price/mg	0.12	0.10–0.15	Gamma (100,3.011)	[23]
Chemotherapy infusion 1 hour	142.55	114.04–171.06	Gamma (100,0.702)	[25]
Chemotherapy infusion additional hour	30.68	24.54–36.82	Gamma (100,2.290)	[25]
**Adverse event management cost**s				
1st-line pembrolizumab plus EP	8680.05	6944.04–10416.06	Gamma (100,0.012)	[26]
1st-line placebo plus EP	8110.70	6488.56–9732.84	Gamma (100,0.012)	[26]
Subsequent therapy of pembrolizumab plus EP group	5429.48	4343.58–6515.38	Gamma (100,0.018)	[26]
Subsequent therapy of placebo plus EP group	6129.80	4903.84–7355.76	Gamma (100,0.016)	[26]
**Other disease management costs**				
Outpatient follow-up visit	52.33	41.86–62.80	Gamma (100,1.911)	[18]
Monthly supportive care	637	509.60–764.40	Gamma (100,0.157)	[24]
Death associated costs	9433	7546.40–11319.60	Gamma (100,0.011)	[24]
**Utilities**				
>12 months prior to death	0.834	0.823–0.846	Beta (3354,668)	[21]
6–12 months prior to death	0.765	0.743–0.786	Beta (1143,351)	[21]
1–6 months prior to death	0.709	0.690–0.728	Beta (1157,639)	[21]
1 month prior to death	0.563	0.461–0.665	Beta (51,37)	[21]
**Others**				
Proportion of carboplatin in first-line treatment	0.711	0.574–0.860	Beta (27,11)	[12]
proportion of subsequent therapy in Pembrolizumab plus EP group	0.529	0.423–0.635	Beta (45,40)	[12]
proportion of subsequent therapy in placebo plus EP group	0.655	0.524–0.786	Beta (33,17)	[12]
Body Weight (kilograms)	70.32	69.71–70.93	Gamma (100,1.422)	[24]
Body Surface Area (meters^2^)	1.79	1.78–1.80	Gamma (100,55.865)	[24]

EP: etoposide-platinum.

## Results

### Base-case analysis

Based on our basic cost-effectiveness analysis for patients with previously untreated ES-SCLC over a 10-year time horizon (Table 3), incremental costs associated with the first-line use of pembrolizumab plus EP versus placebo plus EP were $87,017 ($146,873 vs $59,286), and was primarily composed of drug acquisition costs for first-line treatment, followed by drug acquisition costs for second-line treatment and AE management costs for first-line treatment. Incremental effectiveness associated with the first-line use of pembrolizumab plus EP versus placebo plus EP were 0.22 QALYs (1.01 QALYs vs 0.79 QALYs). Overall, first-line pembrolizumab plus EP produced an ICER of $364,373 per QALY compared to placebo plus EP.

**Table 3:** Base case results.

Outcomes	Pembrolizumab plus EP	Placebo plus EP	Difference
**LYs**	1.43	1.13	0.30
PFS state	0.57	0.45	0.12
PS state	0.86	0.68	0.18
**QALYs**	1.01	0.79	0.22
PFS state	0.46	0.35	0.11
PS state	0.55	0.44	0.11
**Costs, $US**	126,362	44,890	81,472
1st-line drug acquisition costs	94,384	3,600	90,784
2nd-line drug acquisition costs	1,439	614	825
Drug administration cost	8,680	8,111	569
AEs management (first-line)	1,250	978	272
AEs management(second-line)	13,572	23,180	-9,608
Follow-up visit	3,482	4,612	-1,130
Supportive care	817	607	210
Death cost	2,738	3,188	-451
**ICER, $US**			
Per LY			271,574
Per QALY			334,373

EP: etoposide-platinum; LY: life-year; QALY: quality-adjusted life-year; PFS: progression-free survival; PS: progressed survival; ICER: incremental cost-effectiveness ratio.

### Sensitivity analyses

**Fig 2:**
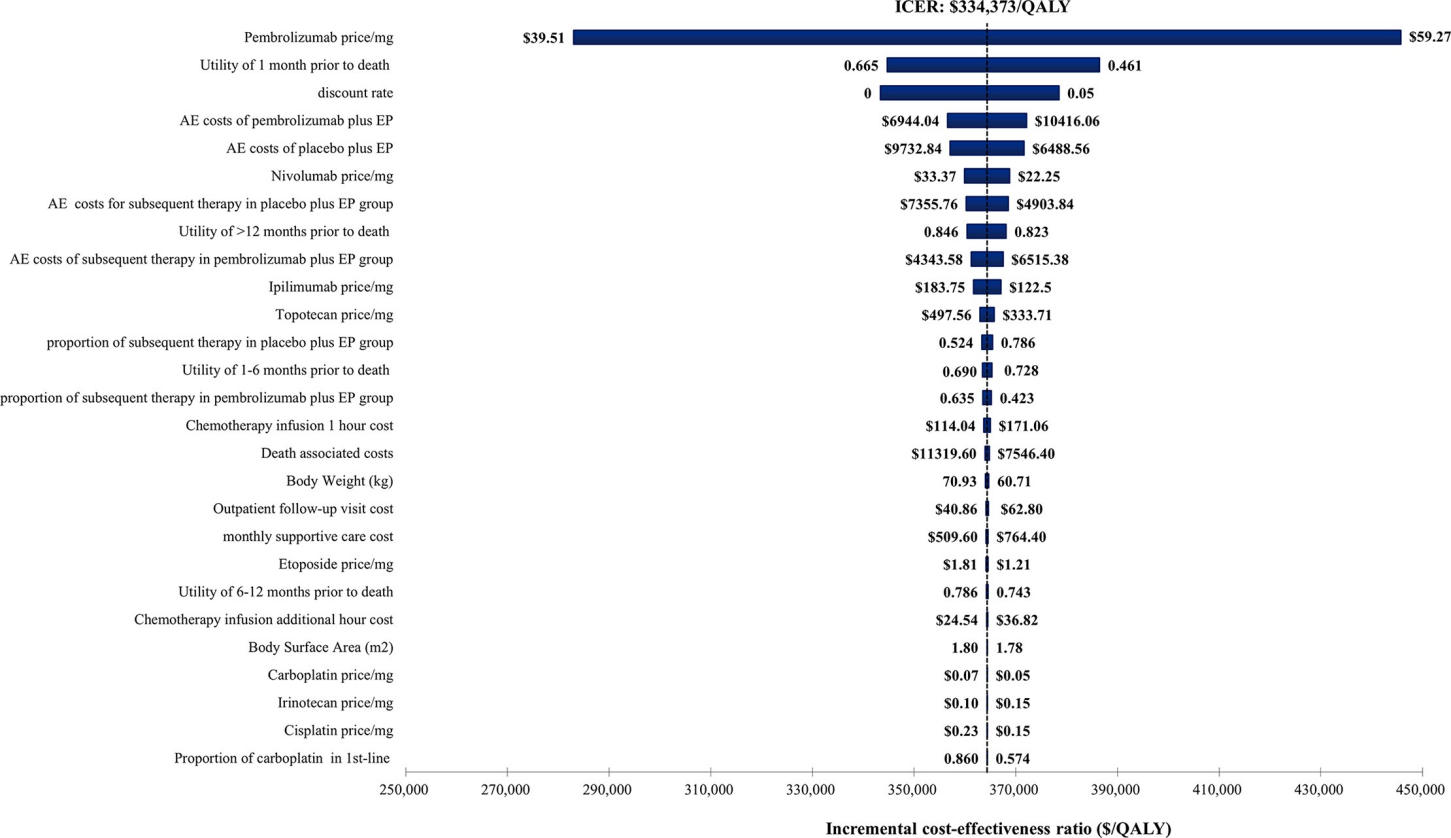
The results of deterministic sensitivity analysis. The black dotted line represents the ICER of $334,373 per quality-adjusted life-year (QALY) from the base case results. The ranges for each model parameters listed represents the lower and upper bounds used in the sensitivity analysis. ICER: incremental cost-effectiveness ratio; EP: etoposide-platinum.

The tornado diagram depicted in Fig 2 reported the results of DSA. The ICER between pembrolizumab plus EP and placebo plus EP decreased significantly with the decreasing price of pembrolizumab and the utility of 1 month prior to death. Other parameters had smaller influences on the ICER for pembrolizumab plus EP vs placebo plus EP. In general, within the upper and lower limits of model parameters, the ICER fluctuated in the range of $280,000 per QALY and $460,000 per QALY, which were much higher than the WTP threshold of $100,000 per QALY in our model.

**Fig 3:**
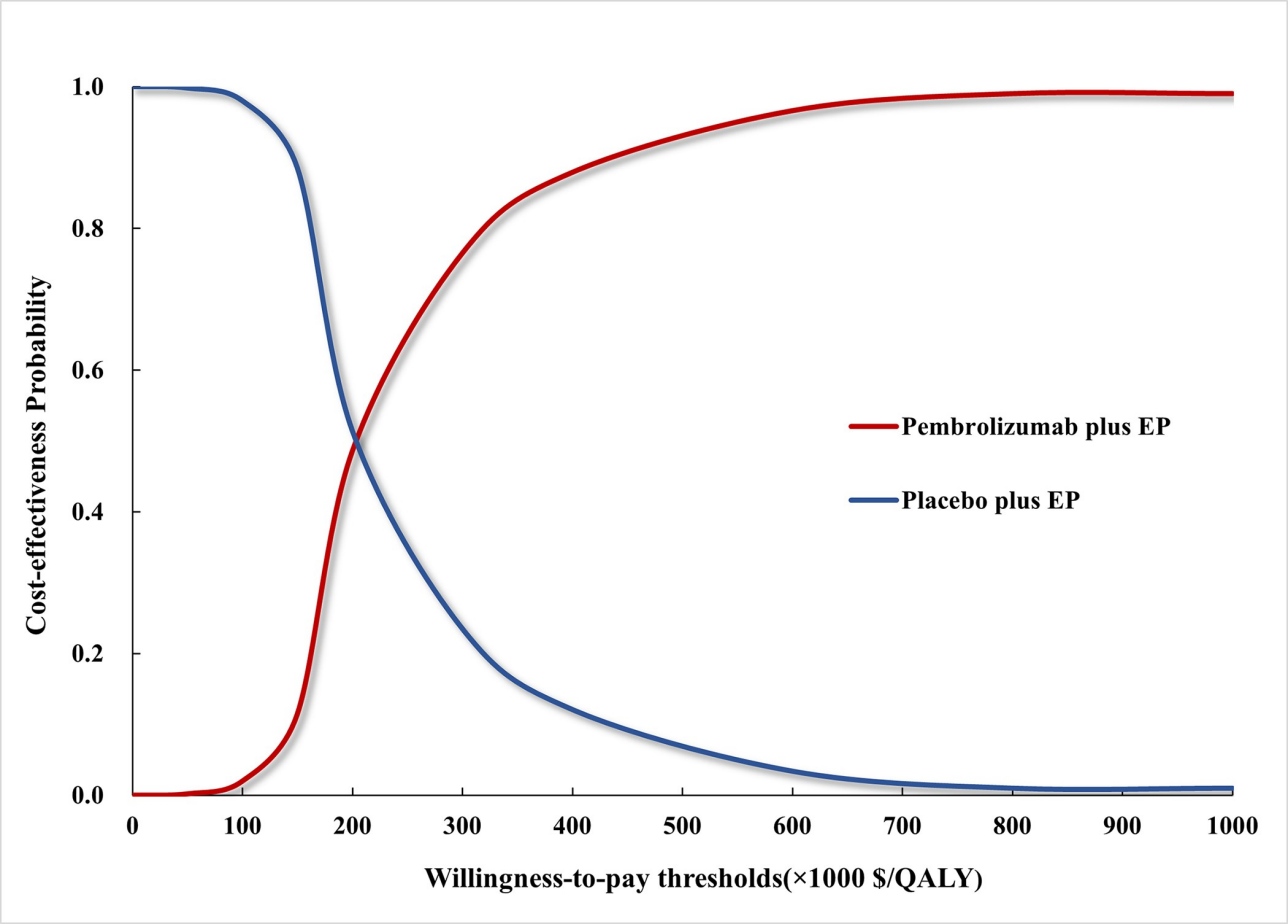
The cost-effectiveness acceptability curve. The red curve indicated the probability of pembrolizumab plus EP being cost-effective against placebo plus EP under different WTP thresholds. QALY: quality-adjusted life-year; EP: etoposide-platinum.

The cost-effectiveness acceptability curve depicted in Fig 3 reported that the cost-effectiveness probability of pembrolizumab plus EP under different WTP thresholds. Compared with placebo plus EP, pembrolizumab plus EP had a 1.55% probability being cost-effective at the WTP threshold of $100,000 per QALY. When the WTP threshold increased to $210,000 per QALY, pembrolizumab plus EP had a 50% probability being cost-effective. In addition, we also analyzed the cost-effectiveness acceptability of pembrolizumab plus EP under different pembrolizumab price (**S5 Fig**). Compared with placebo plus EP, pembrolizumab plus EP had a probability of over 50% being cost-effective, when the price of pembrolizumab per mg was less than $10.

## Discussion

First-line treatment options for ES-SCLC are usually limited to conventional chemotherapy, until the approval of atezolizumab or durvalumab combined with chemotherapy in recent years [10, 11]. A latest phase III clinical study, KEYNOTE-604, compared the star ICI, pembrolizumab plus EP with placebo plus EP in the first-line treatment for ES-SCLC [12]. Preliminary results from the KEYNOTE-604 trail were mixed. Pembrolizumab plus EP did provide a significant clinical benefit in the first-line treatment for ES-SCLC, however the median OS was close to the prespecified significance threshold [12]. Given that medians do not fully capture the PFS and OS benefits of immuotherapy [28]. and the EYNOTE-604 KM curves supported a long-term benefit of pembrolizumab plus EP [12], as well as the price gap between pembrolizumab plus EP and EP, it is significant for us to evaluate its cost-effectiveness from the US payer perspective by simulating the long-term survival of patients with ES-SCLC in KEYNOTE-604 trial through Markov model.

Based on our results, the addition of pembrolizumab to first-line EP chemotherapy produced an ICER of $334,373 per QALY, which was beyond the WTP threshold of $100,000 per QALY. As a result, pembrolizumab plus EP was not considered to be a cost-effective first-line treatment for patients with ES-SCLC from the US payer perspective. DSA demonstrated that the most influential parameter of our model was the price of pembrolizumab. Low price of pembrolizumab would lower the total cost of pembrolizumab plus EP, therefore, incurred a decrease in the ICER. We found that the pembrolizumab plus EP would produce an ICER lower than the WTP threshold when the reduction in the price of pembrolizumab exceeded 65%. Another parameter that has a great influence on our model was the utility of 1 month prior to death. Due to the lack of published health utility value data for ES-SCLC patients, utility values used in this analysis therefore referred to NSCLC patients [20]. To illustrate the influence of health utility values on the model, each utility value was defined a variable range in sensitivity analyses. The results indicated that the upper and lower limits of utility values were failed to make pembrolizumab plus EP cost-effective.

In view of the relatively new clinical evidence of first-line application of immunotherapy in ES-SCLC, its economic evaluation was rarely reported. There are only two published cost-effectiveness analyses for immunotherapy combined with chemotherapy as first-line treatment for ES-SCLC, both from an American perspective [17, 18]. One established a partition survival model to compared durvalumab plus EP versus EP for patients with previously untreated ES-SCLC, and found that durvalumab plus EP was associated with an ICER of $355,488 per QALY. The other study developed a Markov model to assess the cost-effectiveness of atezolizumab plus chemotherapy, and reported an ICER of $528,810 per QALY. Although these two previous studies and our current study concluded that the combination of immunotherapy and chemotherapy was not a cost-effective choice in the first-line treatment of ES-SCLC, pembrolizumab combined with chemotherapy strategy seemed to provide a more effective balance between incremental cost and quality-adjusted survival gained than was atezolizumab or durvalumab.

Strengths of our study were worth highlighting. First, the cost and clinical outcomes associated with pembrolizumab plus EP and placebo plus EP over a 10-year time horizon were estimated through economic modeling. In our model, clinical efficacy and safety data were pooled from the KEYNOTE-604 trial, and costs were mainly collected from Medicare in 2020. Compared with conventional meta-analytic techniques, a Markov model was able to provide more accurate and reliable long-term projection. Second, the dose sizes and infusion timing for subsequent therapy in our model were consistent with the recommendation of NCCN guidelines [19], which may be closer to real clinical practice. Third, the cost of first-line pembrolizumab plus EP treatment for 7 median cycles (range,1–35 cycles) was considered in our model to avoid the influence of the duration of pembrolizumab on the results. As observed in the KEYNOTE-604 trial, patients discontinued treatment not just because of disease progression, but also because of AE, physician decision, radiographic progression, withdrawal of consent. Therefore, the median number of cycles would better reflect the first-line therapy time in the KEYNOTE-604 trial. In addition to the strengths mentioned, our trial-based Markov model simulated the process of treatment and survival for patients with previously untreated ES-SCLC, so as to imitate general clinical treatment scenes. Therefore, our study findings can easily translate into general practice.

Of course, our study has several limitations. First, because quality-of-life data for ES-SCLC patients was unavailable in the KEYNOTE-604 trial, we had to make certain assumptions about the health state utilities. We assumed that the patient’s quality of life was similar to that of that of the patients in the KEYNOTE-189 trial based on the similarity of treatment strategies between the two trials. However, it should be noted that in addition to the different participants recruited in the two clinical trials, the chemotherapy drugs used in the two studies were also different, which may lead to potential bias and uncertainty in our model. Nevertheless, our findings remained robust within the range of health state utilities. Second, there were inherent uncertainty in the long-term extrapolations of PFS and OS. In our model, published KM survival curves were digitized to replicate the PFS and OS data, which cannot be directly obtained in the KEYNOTE-604 trial. Among four most commonly used parametric survival functions, log-logistic distribution was chosen to fit and extrapolate those replicated survival data based on statistical goodness of fit test. Although this method emulated the survival data observed in the keynote-604 trial, the current model could be optimized when more mature data long-term survival data are available. Third, a number of subsequent therapies were available to patients who experienced disease progression, based on the subsequent therapy date from the KEYNOTE-604 trial. To simplify the model, we only considered three common subsequent therapies, nivolumab plus ipilimumab, etoposide, topotecan and irinotecan. However, the results of sensitivity analysis indicated that the proportion of subsequent therapy and related drug costs had less remarkable effects on our model.

## Conclusion

In the first-line treatment of ES-SCLC, adding pembrolizumab to standard chemotherapy would not be a cost-effective option from the US payer perspective. Price reductions remain the most practical solution to balance the incremental cost and quality-adjusted survival gain for pembrolizumab combined with chemotherapy strategy.

## Supporting information

S1 Fig OS data fitted and extrapolated for pembrolizumab plus EP. OS: overall survival; EP: etoposide-platinum.
(TIF)

S2 Fig OS data fitted and extrapolated for placebo plus EP. OS: overall survival; EP: etoposide-platinum.
(TIF)

S3 Fig OS data fitted and extrapolated for pembrolizumab plus EP. PFS: progression-free survival; EP: etoposide-platinum.
(TIF)

S4 Fig OS data fitted and extrapolated for placebo plus EP. PFS: progression-free survival; EP: etoposide-platinum.
(TIF)

S5 Fig The cost-effectiveness acceptability curve. The red curve indicated the probability of pembrolizumab plus EP being cost-effective against placebo plus EP under different pembrolizumab price/mg at the WTP thresholds of $100,000 per QALY. QALY indicated quality-adjusted life-year; EP, etoposide-platinum.
(TIF)

S1 Table Treatment regimens included in the model. EP: etoposide-platinum; AUC: area under the curve.
(DOCX)

S2 Table Survival functions fitted and extrapolated. OS: overall survival; PFS: progression-free survival.
(DOCX)

S3 Table Cost and utility parameters. (DOCX)
