# Supplementary figures and images for: Cost-effectiveness analysis of pembrolizumab plus chemotherapy as first-line therapy for extensive-stage small-cell lung cancer
Source: PLoS One. 2021 Nov 15;16(11):e0258605. doi: 10.1371/journal.pone.0258605 (PMC8592441; doi:10.1371/journal.pone.0258605)

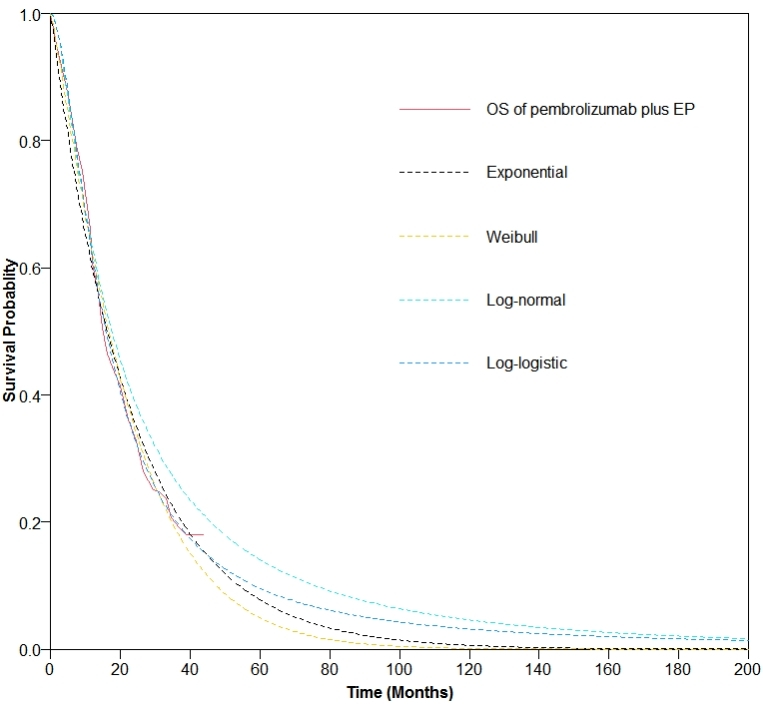

Supplement: S1 Fig — OS: overall survival; EP: etoposide-platinum. (TIF) [file pone.0258605.s001.tif]

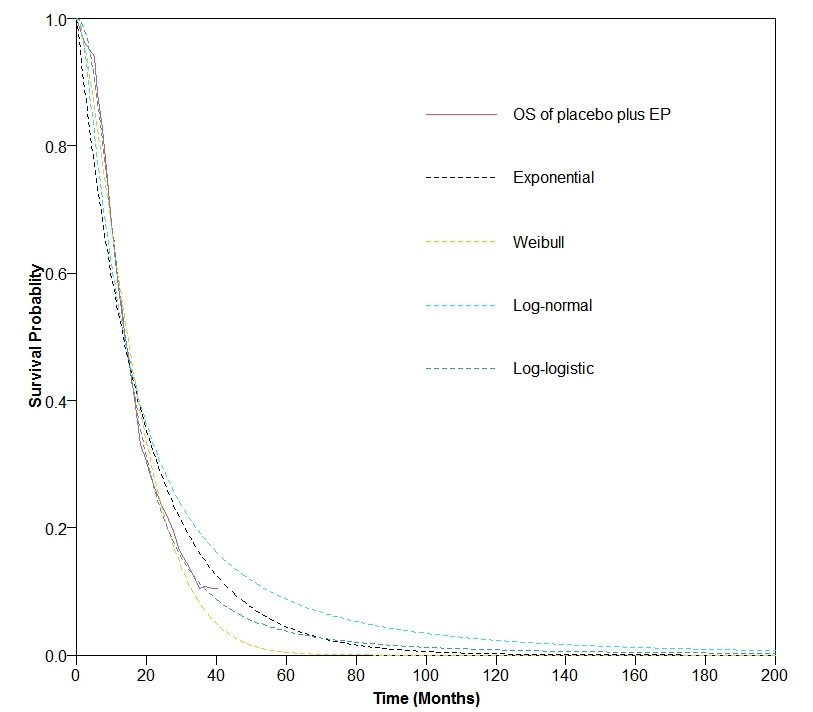

Supplement: S2 Fig — OS: overall survival; EP: etoposide-platinum. (TIF) [file pone.0258605.s002.tif]

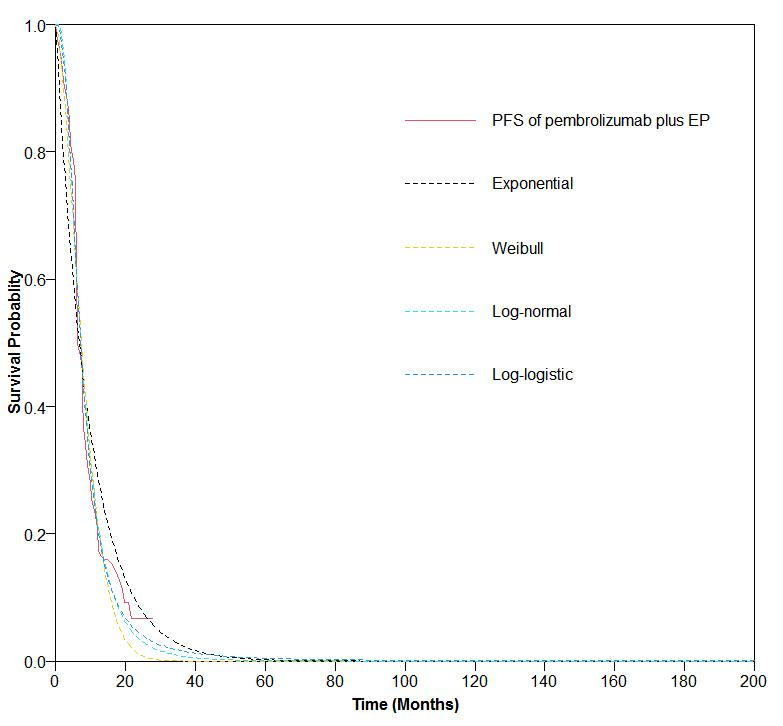

Supplement: S3 Fig — PFS: progression-free survival; EP: etoposide-platinum. (TIF) [file pone.0258605.s003.tif]

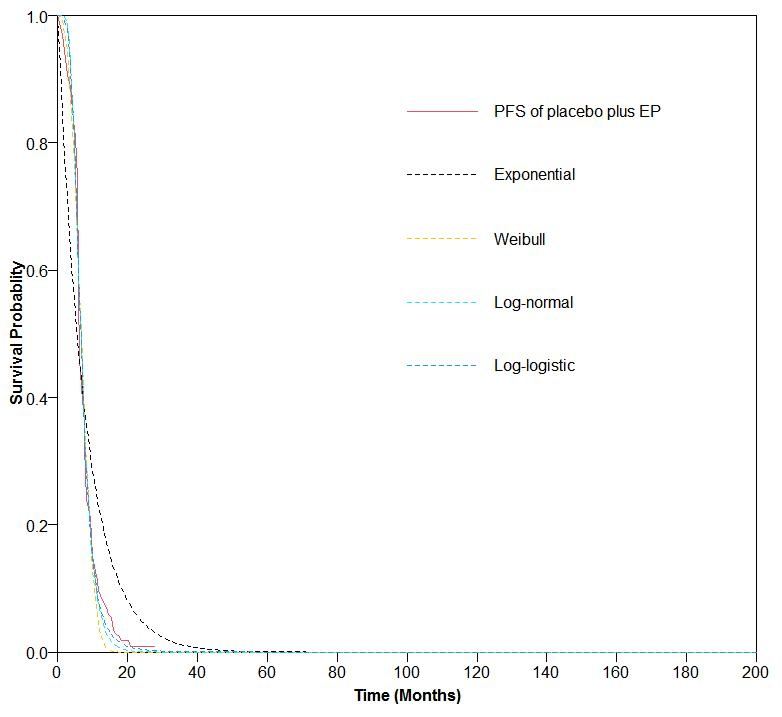

Supplement: S4 Fig — PFS: progression-free survival; EP: etoposide-platinum. (TIF) [file pone.0258605.s004.tif]

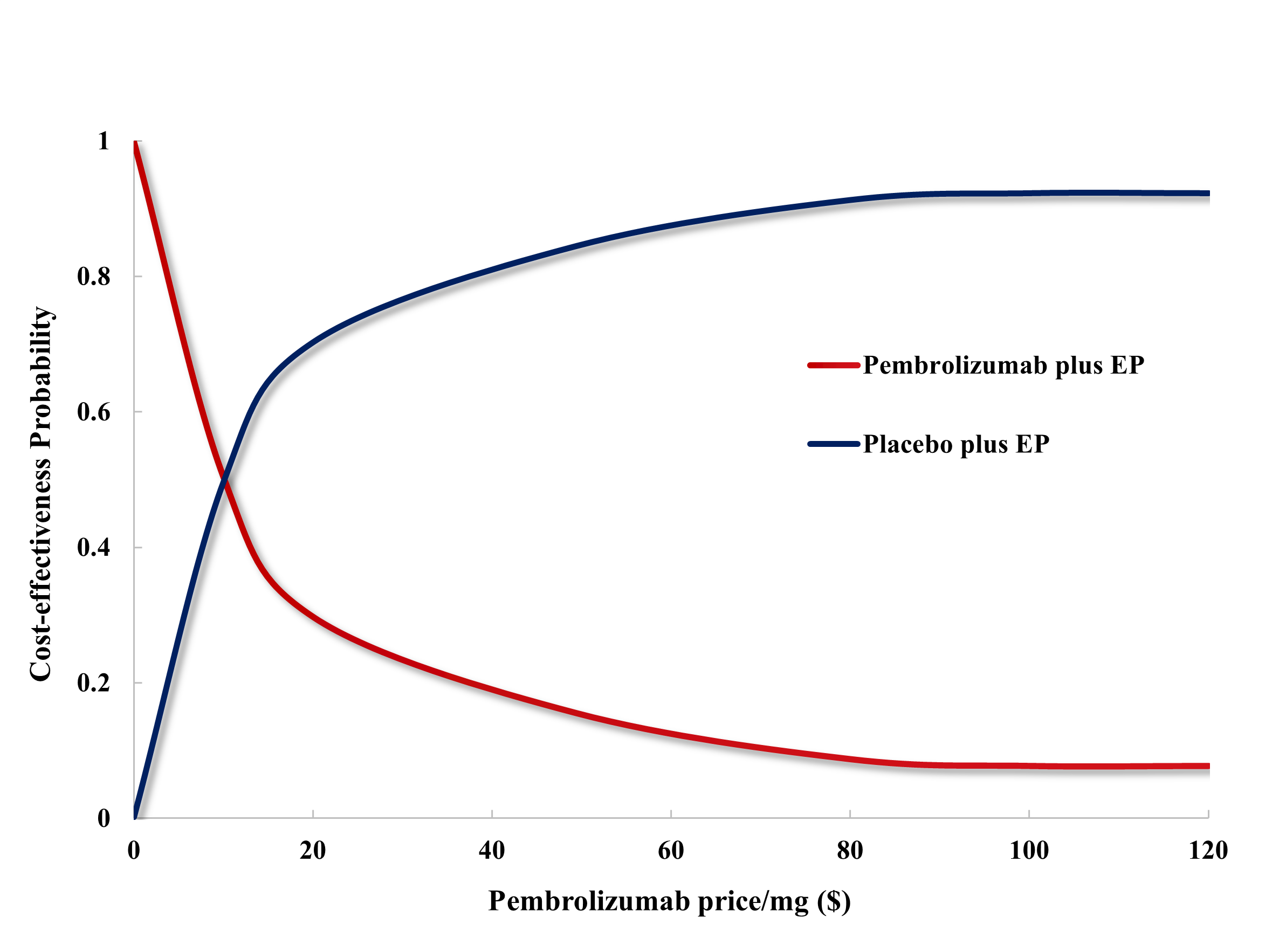

Supplement: S5 Fig — The red curve indicated the probability of pembrolizumab plus EP being cost-effective against placebo plus EP under different pembrolizumab price/mg at the WTP thresholds of $100,000 per QALY. QALY indicated quality-adjusted life-year; EP, etoposide-platinum. (TIF) [file pone.0258605.s005.tif]
