# Supplementary material for: Cost-effectiveness analysis of pembrolizumab plus chemotherapy as first-line therapy for extensive-stage small-cell lung cancer
Source: PLoS One. 2021 Nov 15;16(11):e0258605. doi: 10.1371/journal.pone.0258605 (PMC8592441; doi:10.1371/journal.pone.0258605)
Supplement: S1 Table — EP: etoposide-platinum; AUC: area under the curve. (DOCX) [file pone.0258605.s006.docx]

**S1 Table. Treatment regimens included in the Model**

| **Regimens** | **Dose Sizes** | **Infusion Timing** | **Source** |
| --- | --- | --- | --- |
| 1st-line pembrolizumab plus EP | Pembrolizumab, 200 mg;  Etoposide, 100 mg/m^2^;  Carboplatin, AUC 5.0 mg/ml/min; or cisplatin, 75 mg/m^2^ | Pembrolizumab, 3weeks up to 35 cycles;  EP, 3 weeks for the first 4 cycles | Charles, et al., 2020 |
| 1st-line placebo plus EP | Etoposide, 100 mg/m^2^;  Carboplatin, AUC 5.0 mg/ml/min; or cisplatin, 75 mg/m^2^ | Saline placebo, 3 weeks up for 35 cycles;  EP, 3 weeks for the first 4 cycles | Charles, et al., 2020 |
| 2nd-line nivolumab plus ipilimumab | Nivolumab, 3 mg/kg;  Ipilimumab,1 mg/kg | Nivolumab, 2 weeks;  Ipilimumab, 6 weeks | Hellmann, et al., 2019 |
| 2nd-line etoposide plus carboplatin | Etoposide,100 mg/m^2^;  Carboplatin, AUC 5.0 mg/ml/min | 3 weeks up to 6 cycles | Nathalie, et al., 2020 |
| 2nd-line topotecan | Topotecan, 2.3 mg/m^2^ | 3 weeks up to 6 cycles | Nathalie, et al., 2020 |
| 2nd-line irinotecan | Irinotecan, 50 mg/m^2^;  Carboplatin, AUC 5.0 mg/ml/min | 3 weeks up to 6 cycles | Schmittel, et al., 2006 |

EP: etoposide-platinum; AUC: area under the curve.
