# Supplementary material for: Cost-effectiveness analysis of pembrolizumab plus chemotherapy as first-line therapy for extensive-stage small-cell lung cancer
Source: PLoS One. 2021 Nov 15;16(11):e0258605. doi: 10.1371/journal.pone.0258605 (PMC8592441; doi:10.1371/journal.pone.0258605)
Supplement: S2 Table — OS: overall survival; PFS: progression-free survival. (DOCX) [file pone.0258605.s007.docx]

**Table S2 Survival functions fitted and extrapolated**

| **Survival function** | **Pembrolizumab plus EP** | | | | **placebo plus EP** | | | |
| --- | --- | --- | --- | --- | --- | --- | --- | --- |
|  | OS | | PFS | | OS | | PFS | |
|  | AIC | BIC | AIC | BIC | AIC | BIC | AIC | BIC |
| Exponential | -193.0 | -189.1 | -197.8 | -192.6 | -217.4 | -212.2 | -99.8 | -94.7 |
| Weibull | -228.1 | -222.2 | -280.6 | -272.8 | -389.6 | -381.7 | -287.9 | -280.3 |
| Log-normal | -293.3 | -284.7 | -334.9 | -327.1 | -435.7 | -427.6 | -311.8 | -304.2 |
| Log-logistic | -283.6 | -277.6 | -357.3 | -349.5 | -578.1 | -570.3 | -326.7 | -319.0 |

EP: etoposide-platinum; OS: overall survival; PFS: progression-free survival; AIC: Akaike information criterion; BIC: Bayesian information criterion.
