# Supplementary material for: Cost-effectiveness analysis of pembrolizumab plus chemotherapy as first-line therapy for extensive-stage small-cell lung cancer
Source: PLoS One. 2021 Nov 15;16(11):e0258605. doi: 10.1371/journal.pone.0258605 (PMC8592441; doi:10.1371/journal.pone.0258605)
Supplement: S3 Table — (DOCX) [file pone.0258605.s008.docx]

**Table S3 Cost and Utility Parameters**

| **Parameters** | **Baseline values** | **Source** |
| --- | --- | --- |
| **Cost (2020 $US)** |  |  |
| **Regimen related costs** |  |  |
| Pembrolizumab price/mg | 49.39 | CMS 2020 ASP Drug Pricing Files |
| Etoposide price/mg | 1.51 | CMS 2020 ASP Drug Pricing Files |
| Carboplatin price/mg | 0.06 | CMS 2020 ASP Drug Pricing Files |
| Cisplatin price/mg | 0.19 | CMS 2020 ASP Drug Pricing Files |
| Nivolumab price/mg | 27.81 | CMS 2020 ASP Drug Pricing Files |
| Ipilimumab price/mg | 153.13 | CMS 2020 ASP Drug Pricing Files |
| Topotecan price/mg | 414.63 | CMS 2020 ASP Drug Pricing Files |
| Irinotecan price/mg | 0.12 | CMS 2020 ASP Drug Pricing Files |
| Chemotherapy infusion 1 hour | 142.55 | CMS.gov, HCPCS code 96413 |
| Chemotherapy infusion additional hour | 30.68 | CMS.gov, HCPCS code 96415 |
| **Adverse event management costs** |  |  |
| Neutropenia | 12365 | hcupnet. ahrq.gov |
| Anemia | 7248 | hcupnet. ahrq.gov |
| Thrombocytopenia | 11198 | hcupnet. ahrq.gov |
| Pneumonia | 9057 | hcupnet. ahrq.gov |
| **Other disease management costs** |  |  |
| Outpatient follow-up visit | 52.33 | [Zhang](https://pubmed.ncbi.nlm.nih.gov/?term=Zhang+L&cauthor_id=33344252),et al.,2020 |
| Monthly supportive care | 637.00 | Criss, et al.,2019 |
| Death associated costs | 9433 | Criss, et al.,2019 |
| **Utilities** |  |  |
| >12 months prior to death | 0.834 | Insinga, et al., 2018 |
| 6-12 months prior to death | 0.765 | Insinga, et al., 2018 |
| 1-6 months prior to death | 0.709 | Insinga, et al., 2018 |
| 1 month prior to death | 0.563 | Insinga, et al., 2018 |
